# Levamisole-induced leukocytoclastic vasculitis and neutropenia in a patient with cocaine use: An extensive case with necrosis of skin, soft tissue, and cartilage

**DOI:** 10.1186/1940-0640-7-19

**Published:** 2012-09-24

**Authors:** Natasha Purai Arora, Tania Jain, Ravinder Bhanot, Suganthini Krishnan Natesan

**Affiliations:** 1 Department of Internal Medicine, Wayne State University School of Medicine, Detroit Medical Center, 4201 Saint Antoine Street, Detroit, 48201, MI, USA

**Keywords:** Levamisole, Cutaneous vasculitis, Retiform purpura, Leukocytoclastic vasculitis

## Abstract

Levamisole-induced vasculitis is a relatively new entity in people who use cocaine. We describe a 44-year-old woman with a history of cocaine use who presented with a complaint of a painful rash of 2-3 month’s duration on her extremities, cheeks, nose, and earlobes. She had not experienced fever, weight loss, alopecia, dry eyes, oral ulcers, photosensitivity, or arthralgia. Examination revealed tender purpuric eruptions with central necrosis on her nose, cheeks, earlobes, and extremities. Laboratory investigations revealed neutropenia, an elevated erythrocyte sedimentation rate (ESR), presence of lupus anticoagulant, low complement component 3 (C3), and presence of perinuclear anti-neutrophil cytoplasmic antibody (p-ANCA). A urine toxicology screen was positive for cocaine, and gas chromatography–mass spectrometry was positive for levamisole. Skin biopsy showed leukocytoclastic vasculitis and small vessel thrombosis. Necrotic lesions of the nose led to its self-amputation. Large bullae on the lower extremities ruptured, leading to wound infection and extensive necrosis that required multiple surgical debridements. When necrosis progressed despite debridement, bilateral above-knee amputation of the legs was performed. Once new lesions stopped appearing, the patient was discharged home. Two months later, she had a recurrence related to cocaine use. To the best of our knowledge, this is only the second reported case of levamisole-induced vasculitis that required above-knee amputation.

## Background

According to July 2009 estimates, 69% of the cocaine seized by the US Drug Enforcement Administration (DEA) is adulterated with levamisole [1,2]. Levamisole is a veterinary antihelminthic agent that has recently been linked to vasculitis and neutropenia in people with cocaine use [3,4]. We describe a cocaine user with levamisole-induced necrosis of the skin, soft tissue, and cartilage resulting in nasal amputation, earlobe necrosis, and bilateral above-knee amputation (AKA). To the best of our knowledge, this is only the second case of levamisole-induced vasculitis requiring above-knee amputation to be reported in the English language literature, and it is also one of the first reported cases with laboratory confirmation of levamisole exposure.

### Case presentation

**Figure 1:**
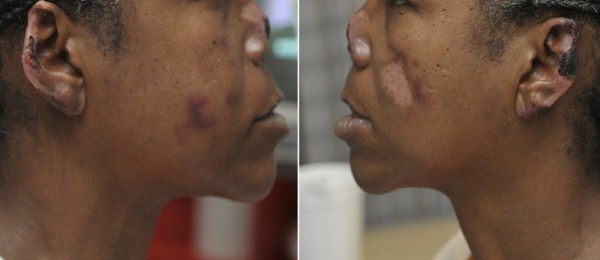
Purpuric lesions and necrosis of both earlobes.

**Figure 2:**
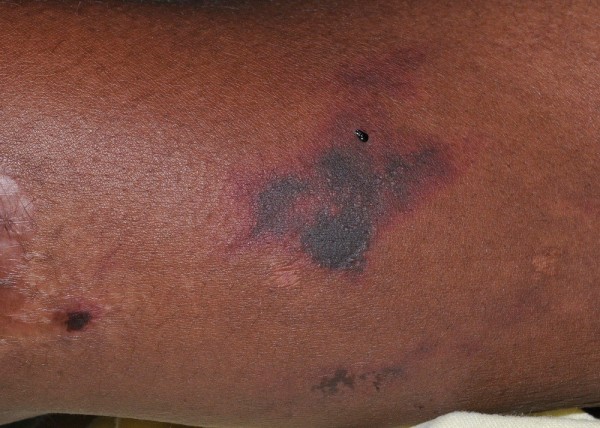
Purpuric patches on extremities.

A 44-year-old African American woman with history of hypertension and asthma presented to the hospital with a complaint of a painful rash on her extremities of 2-3 months’ duration. The rash first appeared on her upper extremities and then progressed to her legs, cheeks, nose, and earlobes. She denied a history of fever, weight loss, alopecia, dry mouth, oral ulcers, painful red eyes, photosensitivity, myalgia, arthralgia, joint swelling, dysphagia, miscarriages, or blood clots. The patient had a chronic history of crack cocaine use and a smoking history of five pack-years. On examination, her vital signs were stable. Skin examination revealed erythematous maculopapular purpuric lesions on her nose, cheeks, and earlobes with central blackish discoloration (Figures 1 and 2). She had several large violaceous plaques and flaccid bullae on her upper and lower extremities and a stage-II ulcer on the medial surface of her right ankle with some serosanginuous discharge. Other physical examination results were normal.

Laboratory investigations revealed leukopenia with a white blood cell count (WBC) of 2,600 cells/μL, neutropenia with an absolute neutrophil count (ANC) of 900 cells/μL, an elevated erythrocyte sedimentation rate (ESR) of 47 mm/hour, presence of lupus anticoagulant, low complement component 3 (C3) (85 mg/dL), a normal coagulation profile, absence of antinuclear antibody (ANA), absence of cardiolipin antibody, presence of perinuclear anti-neutrophil cytoplasmic antibody (p-ANCA) against myeloperoxidase (MPO), and absence of antiproteinase 3 (anti-PR3) antibody.

**Figure 3:**
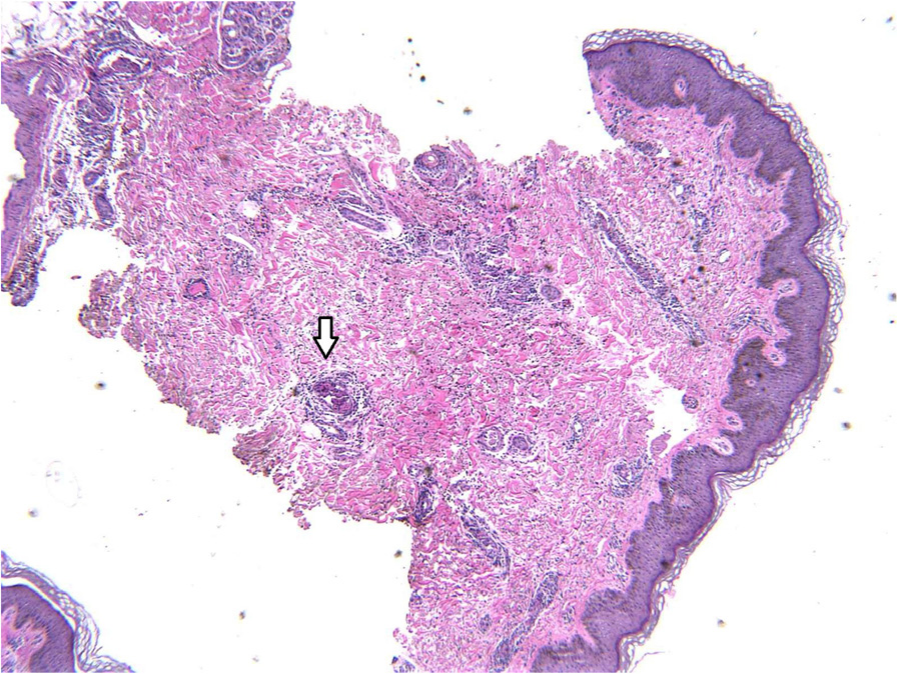
Hematoxylin & eosin staining of skin biopsy sample showing leuckocytoclastic vasculitis with angiocentric infiltrates of mixed inflammatory cells (white arrow) and small vessel thrombosis.

**Figure 4:**
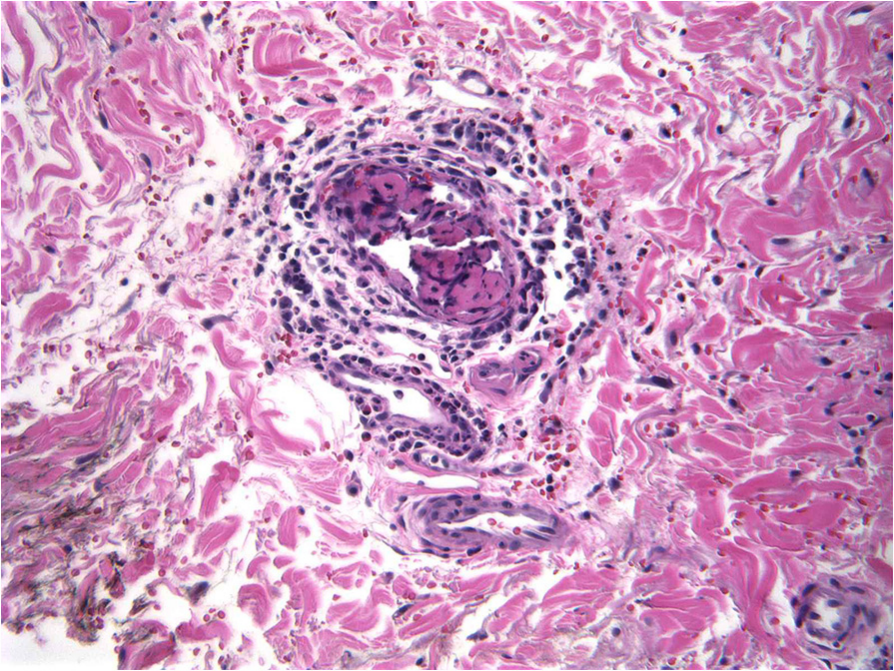
**Leukocytoclastic vasculitis.** A dermal small vessel showing a neutrophilic-rich infiltrate with karyorrhexis and extravasated erythrocytes within the vessel wall and adjacent tissue. Intra-luminal fibrin thrombi are also present.

Her urine toxicology screen was positive for cocaine, and gas chromatography–mass spectrometry (GCMS) was positive for levamisole. Punch biopsy of the skin from involved areas showed leukocytoclastic vasculitis with angiocentric infiltrates of mixed inflammatory cells and small vessel thrombosis with multiple fibrin thrombi in the lumen of the vessels (Figures 3 and 4).

**Figure 5:**
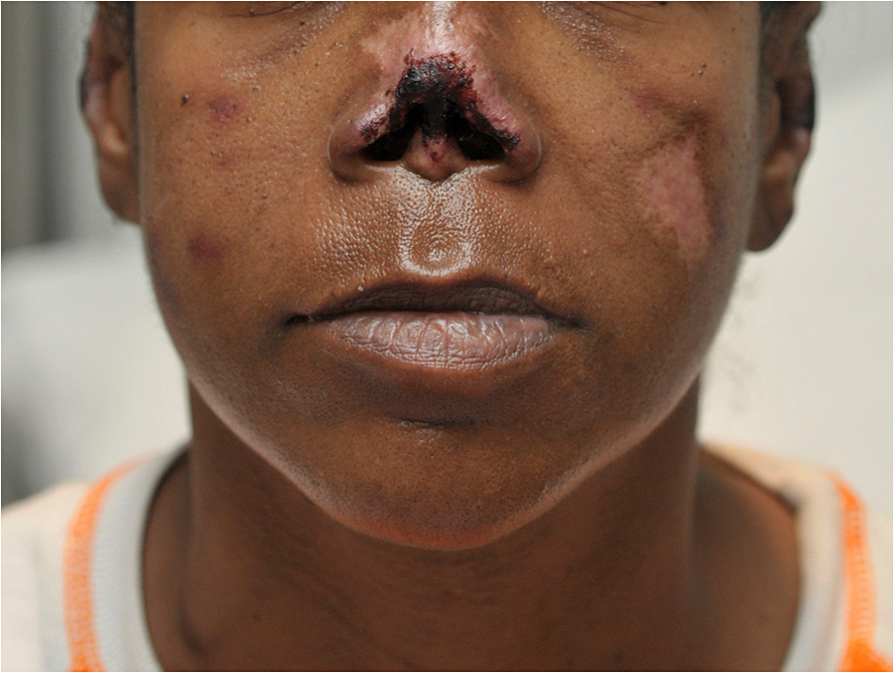
Necrotic lesions on the nose that eventually led to nasal septal destruction and self-amputation.

Lesions on the lower extremities continued to expand, and those on nose, left cheek, and ears became necrotic. Large lower extremity bullae ruptured, leading to wound infection and septicemia with *Staphylococcus aureus*, *Enterobacter cloacae,* and *Morganella morganii,* which were treated with broad-spectrum antibiotics. At this point, skin involvement was estimated to be 35% of the total body surface area, and the patient was managed in a burn unit. Extensive and infected necrosis of the lower extremities required fascial excision of the dead skin and subcutaneous tissue. The patient underwent two further surgical debridements. Despite this, necrosis progressed, and attempts at nonoperative management were not successful. Eventually, bilateral AKA of the legs above the areas of necrotic skin was performed to control the spread of nonhealing necrotic wounds. The decision to proceed with bilateral AKA was based on the presence of a significant degree of necrosis of leg muscles and the poor functional prognosis for the lower extremities, even if the nonhealing wounds eventually closed with local wound care or skin grafts. Necrotic lesions on the nose led to its self-amputation (Figure 5). The patient’s pain was controlled with morphine (patient controlled analgesia). New lesions stopped appearing a few days following surgery, and she was discharged home with a prescription of gabapentin for neuropathic pain.

**Figure 6:**
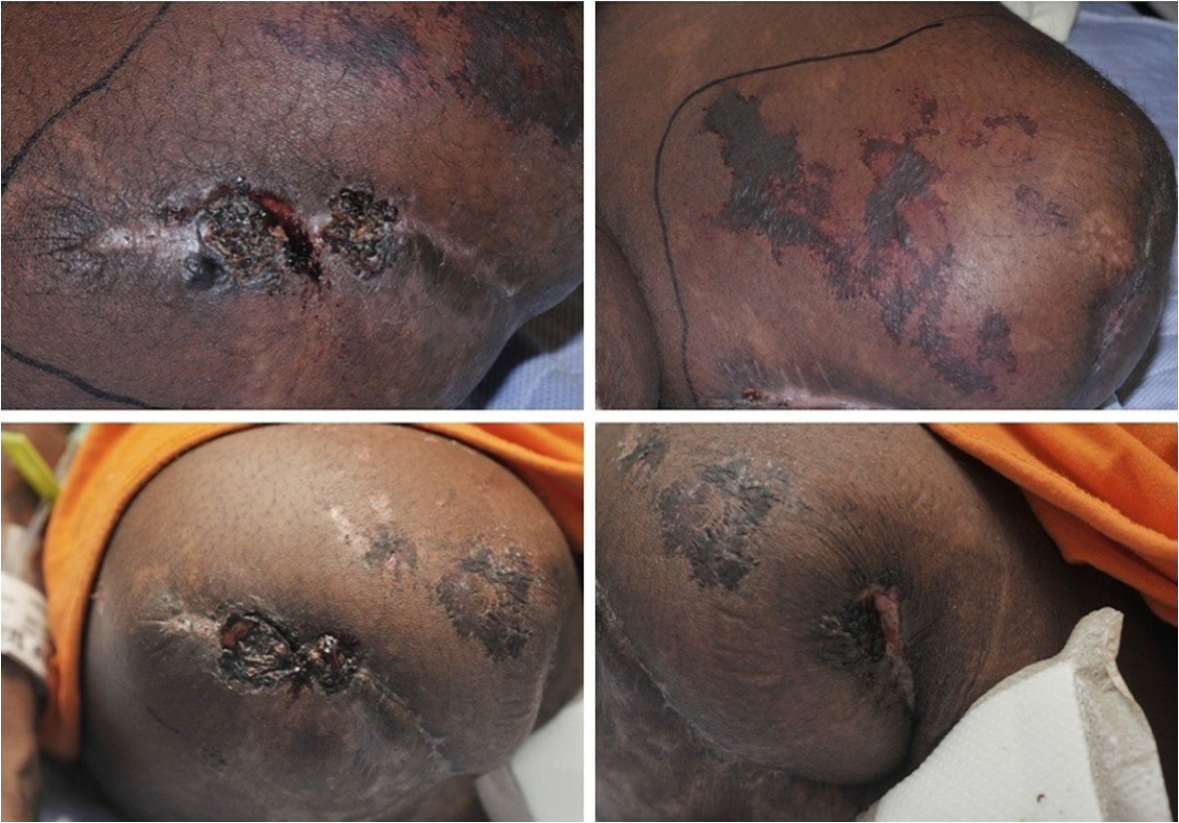
Recurrent purpuric and necrotic lesions on the amputation stumps with repeat use of cocaine.

Two months later, the patient was readmitted with new painful necrotic lesions on the amputated stumps (Figure 6). She admitted to cocaine use three days prior to this admission. She was treated with intravenous methylprednisolone for three days followed by tapering doses of oral prednisone for seven days. Her pain and lesions improved significantly, and she was discharged home with a plan to follow up with the hospital’s plastic surgery service for nose reconstruction.

## Discussion

Levamisole is a veterinary antihelminthic agent currently used to treat worm infestations in cattle, sheep, and pigs [5]. In the past, it was used in humans to treat various autoimmune disorders and cancers because of its immunomodulatory properties [5]. Levamisole-induced vasculitis (LIV) was first reported in a rheumatoid arthritis patient in 1978 [6]. Since 2009, levamisole has also been linked to cutaneous vasculitis in people who use cocaine.

To the best of our best knowledge, 32 cases of LIV in patients with cocaine use have been reported to date [3,4,7-22]. Clinical features, laboratory results, skin biopsy findings, and the treatment of these patients are summarized in Tables 1, 2 and 3. Of the 32 patients described, only eight had levamisole exposure confirmed by urinalysis or GCMS, although other authors speculated on this link based on the presence of levamisole in approximately 69% of the cocaine entering the United States. Among all the reported cases, only one (published twice by different authors, i.e., Ching et al. [15] and Mouzakis et al. [16]) had extensive necrosis requiring AKA. The patient was a 54-year-old man positive for p-ANCA who developed fever, agranulocytosis, and extensive skin necrosis following heavy intranasal cocaine use. Necrosis was present on more than 50% of his total body surface area, requiring thorough wound debridement, skin grafting, and left-sided AKA. Unlike our patient, AKA in this case was unilateral, and levamisole exposure was not confirmed by GCMS since the patient presented late after the likely exposure. The authors speculated that levamisole exposure was probable based on the patient’s clinical presentation and history of cocaine use. Our patient represents the most extensive confirmed case of LIV with involvement of 35% of her total body surface area.

**Table 1:** Clinical features of all reported cases of levamisole-induced cutaneous vasculitis in patients with cocaine use

**Authors**	**Age/Sex**	**Clinical findings**
Gross RL et al. [3]	50 years/M	Retiform pupuric plaques with ulceration on earlobes/helix and lower extremities
	42 years/M	Reticulate pink/purple erythematous eruptions on legs, chest, arms, and back
	42 years/F	Violaceous papules, reticular-purpuric plaques on arms, breasts, legs, and ears
	59 years/F	Purpuric, necrotic plaques and bullae on face, ears, arms, right cheek, chest, and axilla
Ullrich K et al. [4]	45 years/M	Painful necrotic purpura and skin nodules on extremities and ears
	49 years/F	Painful purpura on extremities, trunk, and earlobes
	27 years/F	Painful purpura on lower extremities, arthralgia
	29 years/F	Purpuric lesion with central necrosis on left foot, purpuric lesions on both ears
	55 years/F	Episodic rash on face, trunk, and extremities
Bradford M et al. [7]	57 years/F	Purpuric plaques with central necrosis on cheeks and earlobes
	22 years/F	Erythematous plaques with central necrosis on face, ears, legs, thighs, and buttocks
Buchanan JA et al. [8]	Not specified/M	Purple discoloration on left upper arm and right second toe, bilateral necrosis of ears
Walsh NMG et al. [9]	39 years/F	Retiform purpura, hemorrhagic bullae on legs, trunks, and buttocks
	49 years/F	Retiform purpura on chest, limbs, bilateral nasal mucosal ulcers
Waller JM et al. [10]	38 years/F	Retiform purpura with central necrosis on earlobes, cheeks, breast, extremities
	43 years/F	Retiform purpura with central necrosis on legs, arms, and pinna; livedo racemosa, splinter hemorrhages
Farhat EK et al. [11]	43 years/F	Retiform purpura with central necrosis on bilateral lower extremities
	41 years/F	Painful retiform purpuric patches on thighs, buttocks, trunk, upper extremities, and nasal tip
Click J [12]	29 years/F	Calf ulcerations, retiform purpura
Geller L et al. [13]	50 years/F	Stellate purpura with central necrosis on upper extremities, ears, back, and breasts
Han C et al. [14]	52 years/F	Painful retiform purpura with ulceration on arms, legs, nasal tip, cheeks, ears
Ching J et al. [15]/ Mouzakis J et al. [16]	54 years/F	Purpuric patches and plaques on legs, upper extremities, perinasal area, anterior trunk, face, and ears
Jacob RS et al. [17]	41 years/F	Tender purpuric patches and plaques on ears, legs, upper extremities, back, toes, and soles
	48 years/F	Tender purpuric patches and plaques on ears and left cheek
Lung D et al. [18]	44 years/F	Stellate, purpuric macules and plaques with central necrosis on legs, abdomen, and face
Zwang NA et al. [19]	52 years/M	Violaceous retiform papules/plaques on arms, legs, back, trunk, scalp, buttocks, fingers, foot, and ear
Chung C et al. [20]	46 years/F	Purpura and necrosis on bilateral ears, cheeks, and upper and lower extremities
	46 years/F	Bilateral ear necrosis, diffuse retiform purpura with necrosis on neck, trunk, and extremities
	37 years/M	Tender bilateral ear purpura and necrosis, diffuse retiform purpura on trunk and extremities
	50 years/M	Tender purpura and necrosis on both ears, purpura and bullae on trunk and extremities
Jenkins J et al. [21]	47 years/M	Painful retiform purpura with necrotic center on legs, ears, cheek, tongue, trunk, and genitalia
John S et al. [22]	52 years/F	Painful violaceous plaques and flaccid bullae on right ankle, legs, left arm, and left breast
Arora et al. [current report]	44 years/F	Erythematous purpuric lesions with central necrosis on nose, cheeks, earlobes, and extremities

**Table 2:** Laboratory findings of all reported cases of levamisole-induced cutaneous vasculitis in patients with cocaine use

**Authors**	**Age/Sex**	**Leucopenia and/or Neutropenia**	**Antibodies present**
Gross RL et al. [3]	50 years/M	Yes, WBC -3,300/μL, ANC -2400/μL	p-ANCA, ANA, LAC, c-ANCA, anti-ds-DNA
	42 years/M	Yes, WBC -3,200/μL, ANC - 800/μL	p-ANCA, ANA, LAC, c-ANCA, IgM ACL
	42 years/F	No	p-ANCA, c-ANCA, ANA
	59 years/F	No	p-ANCA, ANA, anti-RNP
Ullrich K et al. [4]	45 years/M	Yes, WBC -1,900/μL, ANC -1,000/μL	p-ANCA, c-ANCA, ANA, IgM ACL
	49 years/F	Yes, WBC -3,500/μL, ANC – 0 (zero)	ANA, c-ANCA
	27 years/F	Yes, WBC -1,800/μL, ANC -400/μL	p-ANCA, c-ANCA, IgM ACL, ANA
	29 years/F	No	p-ANCA, c-ANCA, IgM ACL, ANA
	55 years/F	Yes, WBC -3,700/μL, ANC -2,300/μL	p-ANCA, c-ANCA, IgM ACL
Bradford M et al. [7]	57 years/F	Yes, ANC -500/μL	p-ANCA, IgM ACL
	22 years/F	Yes, ANC < 1,000/μL	p-ANCA, IgM ACL
Buchanan JA et al. [8]	Not specified/M	Yes, WBC -1,900/μL	Not reported
Walsh NMG et al. [9]	39 years/F	No	p-ANCA, c-ANCA, ANA, LAC, anti-HNE
	49 years/F	Yes, WBC -2,000/μL, ANC -400/μL	p-ANCA, c-ANCA, ANA, anti-HNE
Waller JM et al. [10]	38 years/F	Yes, ANC -550/μL	p-ANCA, c-ANCA, LAC
	43 years/F	Yes, ANC -560/μL	p-ANCA, IgM ACL, LAC, anti-ds-DNA,
Farhat EK et al. [11]	43 years/F	No	p-ANCA
	41 years/F	No	p-ANCA, IgM ACL
Click J [12]	29 years/F	No	p-ANCA, c-ANCA, ANA
Geller L et al. [13]	50 years/F	Yes, WBC -2,700/μL, ANC -1,400/μL	p-ANCA, IgM ACL
Han C et al. [14]	52 years/F	Yes, WBC -2,400/μL, ANC -1,400/μL	p-ANCA, c-ANCA, IgM ACL
Ching J et al. [15]/ Mouzakis J et al. [16]	54 years/F	Yes, WBC -3,900/μL, ANC -300/μL	p-ANCA, IgM ACL
Jacob RS et al. [17]	41 years/F	Yes, WBC -1,100/μL, ANC -670/μL	p-ANCA, c-ANCA, IgM ACL, ANA, anti-ds-DNA,anti-HNE
	48 years/F	Yes, WBC -800/μL, ANC -300/μL	p-ANCA, c-ANCA, IgM ACL, ANA, anti-ds-DNA,anti-HNE
Lung D et al. [18]	44 years/F	Yes, ANC -270/μL	Not reported
Zwang NA et al. [19]	52 years/M	No	p-ANCA, c-ANCA, IgM ACL, ANA, LAC, anti-HNE, anti-ds-DNA,
Chung C et al. [20]	46 years/F	No	p-ANCA, c-ANCA, IgM ACL
	46 years/F	Yes, WBC and ANC not specified	p-ANCA, c-ANCA, IgM ACL
	37 years/M	No	p-ANCA, ANA
	50 years/M	No	p-ANCA, ANA
Jenkins J et al. [21]	47 years/M	No	p-ANCA
John S et al. [22]	52 years/F	Yes, WBC -2,900/μL, ANC -638/μL	p-ANCA +, anti ds-DNA+
Arora et al. [current report]	44 years/F	Yes, WBC -2,600/μL, ANC -900/μL	p-ANCA+, LAC+

Abbreviations: *M* = male, *F* = female, *WBC* = white blood cell count, *ANC* = absolute neutrophil count, *p-ANCA* = perinuclear anti-neutrophil cytoplasmic antibody, *c-ANCA* = cytoplasmic anti-neutrophil cytoplasmic antibody, *IgM ACL* = immunoglobulin-M anticardiolipin antibody, *ANA* = antinuclear antibody, *LAC* = lupus anti-coagulant, anti-*HNE* = anti-human neutrophil elastase antibody, anti-*ds-DNA* = anti-double-stranded deoxyribonucleic acid antibody, anti-*RNP* = anti-ribonucleoprotein antibody.

**Table 3:** Skin biopsy findings, treatment, and response to treatment information of all reported cases of levamisole-induced cutaneous vasculitis

**Authors**	**Age/Sex**	**Pathology**	**Treatment**	**Response**
Gross RL et al. [3]	50 years/M	Vasculitis, thrombosis, necrosis	Oral prednisone, surgical debridement	Lesions remained stable, developed auditory hallucinations & superinfection
	42 years/M	Small-vessel vasculitis	Supportive care	Lesions improved without any intervention
	42 years/F	Lekocytoclastic vasculitis, thrombosis	Oral prednisone	Lesions improved in one week
	59 years/F	Small-vessel vasculitis, thrombosis	Oral prednisone	Lost to follow-up
Ullrich K et al. [4]	45 years/M	Not reported	Oral prednisone	Initially improved, but symptoms recurred with attempts to taper the steroid dose
	49 years/F	Thrombosis, perivascular neutrophils, and karyorrhetic debris	Supportive, G-CSF	Resolution of lesions with abstinence from cocaine
	27 years/F	Lekocytoclastic vasculitis, thrombosis	Oral prednisone	Lesions resolved with abstinence, arthralgia and neutropenia improved rapidly with prednisone
	29 years/F	Not reported	Supportive care, oral steroids	Lesions resolved with abstinence, septal perforation & arthralia improved with steroids
	55 years/F	Lekocytoclastic vascultis, thrombosis	Steroids, cyclophosphamide	Rapid resolution of lesions
Bradford M et al. [7]	57 years/F	Intravascular thrombosis, no vasculitis	Filgrastim for neutropenia	Lesions resolved spontaneously, neutropenia improved with filgrastim
	22 years/F	Leucocytoclastic vasculitis, thrombosis	Steroids	Lesions and neutropenia improved rapidly
Buchanan JA et al. [8]	Not specified/M	Not done	Subcutaneous phentolamine to both ears	No improvement
Walsh NMG et al. [9]	39 years/F	Thrombosis, leucocytoclastic vasculitis	Anticoagulation, skin debridement and grafting	No new lesions with discontinuation of cocaine, skin lesions healed after multiple full thickness skin grafts
	49 years/F	Thrombosis, no evidence of vasculitis	Supportive care	Lesions improved, recurrences with cocaine use
Waller JM et al. [10]	38 years/F	Leukocytoclastic vasculitis, thrombosis	Supportive care	Lesions improved, recurrences with cocaine use
	43 years/F	Thrombosis of dermal vessels	Supportive care	Lesions improved, recurrences with cocaine use
Farhat EK et al. [11]	43 years/F	Extensive thrombosis, no vasculitis	Supportive care	Not specified
	41 years/F	Thrombosis with no vasculitis	Not specified	Not specified
Click J [12]	29 years/F	Subepidermal bullous dermatitis, lymphocytic perivascular infiltrate	Skin grafts	Lesions were healing well after 4 months
Geller L et al. [13]	50 years/F	Lecocytoclastic vasculitis, thrombosis	Not specified	Not specified
Han C et al. [14]	52 years/F	Thrombotic vasculopathy, no vasculitis	Steroids (iv & oral), dalteparin, warfarin	Lesions and neutropenia improved, had recurrences in with repeat cocaine use, which improved with oral prednisone
Ching J et al. [15]/ Mouzakis J et al. [16]	54 years/F	Small vessel thrombosis, perivascular mononuclear infiltrates	IV steroids, left AKA, skin debridement, allografts	Developed extensive skin necrosis requiring debridement and skin grafts
Jacob RS et al. [17]	41 years/F	Epidermal necrosis, vascular thrombosis, leukocytoclasis	Oral prednisone	Resolution of the majority of the patient’s lesions
	48 years/F	Lymphocytic infiltrate, occlusive vasculopathy, neovascularization	Oral prednisone	Lesions improved
Lung D et al. [18]	44 years/F	Extensive thrombotic vasculopathy	Not specified	Not specified
Zwang NA et al. [19]	52 years/M	Leukocytoclastic vasculitis	Oral prednisone	Lesions healed completely in 3 weeks
Chung C et al. [20]	46 years/F	Small-vessel vascultits with thrombosis	Steroids	Initial improvement, lost to follow-up
	46 years/F	Multiple Intravascular thrombi	IV methyl-prednisolone	Gradual improvement initially, lost to follow-up
	37 years/M	Leucocytoclastic vasculitis	Supportive	Rapid improvement of skin lesions
	50 years/M	Leucocytoclastic vasculitis, panniculitis	Supportive, antibiotics	Rapid improvement of skin lesions
Jenkins J et al. [21]	47 years/M	Leucocytoclastic vascultits, occlusive vasculopathy	Oral & topical steroids, aspirin, pentoxifylline	Lesions resolved over 3 months
John S et al. [22]	52 years/F	Thrombotic vasculopathy, no vasculitis	Supportive, surgical debridement	Had new lesions with repeat cocaine use and necrotic ulceration of old lesions requiring surgical debridement
Arora et al. [current report]	44 years/F	Leucocytoclastic vasculitis, thrombosis	Surgical debridement, pain control, AKA, IV methyl-prednisolone for recurrent lesions	Recurrent lesions improved significantly with IV methylprednisolone

Abbreviations: *M* = male, *F* = female, *AKA* = above knee amputation, *IV* = intravenous.

Levamisole was an FDA-approved drug but was withdrawn for use in humans in the USA in 1999 due to reports of serious adverse effects such as agranulocytosis, thrombocytopenia, arthritis, and LIV [23-26]. However, it is still available for animal use in the United States, Canada, and South America [26]. In the presence of alternative and more efficacious veterinary antihelminthics, such as ivermectin [27,28], the reasons for continued availability of levamisole for animal use are poorly understood, especially in light of the emerging data on the potential dangers of its addition to cocaine.

The practice of adulterating cocaine with levamisole has increased significantly in recent years [26,29]. Several theories exist to explain the reasons for adulterating cocaine with levamisole. One explanation may be levamisole’s ability to potentiate the psychotropic effects of cocaine [26]. Stimulant effects of cocaine are mediated by the blockage of presynaptic reuptake pumps for the monoamine neurotransmitters dopamine, norepinephrine, and serotonin in the central and peripheral nervous systems leading to their enhanced activity [30,31]. Animal data suggest that levamisole may have an inhibitory action on monoamine oxidase and catechol-O-methyltransferase, the enzymes that metabolize catecholamine neurotransmitters [26]. Therefore, levamisole may potentially inhibit the degradation of these stimulatory neurotransmitters, prolonging the duration of their action and adding to the reuptake-inhibition effect of cocaine. Clinically, this may result in enhanced psychotropic effects [26]. Antihelminthic properties of levamisole are due to its species-specific agonistic action at nicotinic acetylcholine receptors of the muscle cells of nematodes [32]. Cocaine may also act on the nicotinic acetylcholine receptors of humans, resulting in increased dopaminergic reuptake inhibition and glutamatergic activity [33]. Although unlikely due to species-specific action of levamisole, it is theoretically possible that cocaine and levamisole may have a synergistic action at nicotinic acetylcholine receptors resulting in increased nicotinic and dopaminergic effects [26]. Also, studies in horses have suggested that levamisole may get metabolized to aminorex, an amphetamine derivative with stimulant effects similar to cocaine and amphetamine [34]. Other possible explanation for using levamisole as a cocaine adulterant may be its use as a “marker” or “signature” compound by manufacturers to trace its market distribution [26]. Some media reports suggest that levamisole is used as a cutting agent for cocaine because it adds bulk and weight to powdered crack cocaine while retaining the appearance and look of pure cocaine, and it also has the ability to pass cocaine purity tests used by drug dealers. Levamisole-induced cutaneous vasculitis has been reported both with smoked crack cocaine [17] and inhaled powdered cocaine [21], indicating that both are adulterated with levamisole.

In addition to levamisole, other commonly used cocaine adulterants include local anesthetics, sugars, stimulants (such as caffeine, ephedrine, phenylpropanolamine, and amphetamines); toxins (such as quinine and strychnine); and inert compounds [35]. Among these cocaine adulterants, only stimulants have been associated with vasculitis upon chronic use. Unlike LIV, the vasculitis associated with chronic stimulant use is usually cerebral or systemic in distribution [35].

As LIV is usually associated with the generation of autoantibodies such as p-ANCA, ANA, and lupus anticoagulant, it may be difficult to differentiate it from autoimmune disorders such as Wegener’s granulomatosis and other small-vessel vasculitides. The exact pathogenic mechanisms responsible for the formation of these autoantibodies remain elusive. Recent reports have suggested that, due to its ability to act as a hapten, levamisole may cause increased formation of antibodies to various antigens and therefore lead to an immune response involving the opsonization and eventual destruction of the leukocytes [33].

Before the recognition of levamisole as an adulterant in the cocaine supply, cocaine alone was associated with an p-ANCA-positive pseudovasculitis in some previous reports [36]. Although clinical presentation and laboratory findings of pseudovasculitis may be similar to true vasculitis, biopsy specimens in pseudovasculitis patients do not reveal the typical histopathologic findings seen in patients with true vasculitis [36]. Moreover, there is a possibility that these cocaine-related pseudovasculitis cases were actually caused by unrecognized contamination with levamisole.

Adulteration of the majority of the cocaine supply entering United States with levamisole is concerning, especially in view of the high frequency of cocaine use in this country. According to the August 2005 National Survey on Drug Use and Health (NSDUH) Report, more than 5.9 million (2.5%) persons aged 12 years or older used cocaine in 2002-2003 [37].

Based on published reports, Levamisole-induced cutaneous vasculitis in cocaine users is more commonly seen in women [3]. Clinical features commonly include a tender purpuric rash in a retiform/reticular distribution with or without necrosis [3]. In addition to leukopenia and neutropenia, laboratory results are usually positive for different types of auto-antibodies such as c-ANCA, p-ANCA, ANA, and lupus anticoagulant [3]. Recurrence or exacerbation of skin lesions with cocaine use have been reported in some cases. Skin biopsy shows either a mixed pattern of leukocytoclastic and thrombotic vasculitis or an isolated thrombotic vasculopathy [3].

Cessation of cocaine use and supportive care of LIV-related skin lesions lead to resolution of symptoms in most of the cases. Steroids have been used in a significant number of previously reported cases with a variable response. The recurrent lesions in our patient improved significantly after intravenous methylprednisolone. However, due to the risk of increased susceptibility to superimposed infections, steroid use should be limited to more severe cases that fail to respond to supportive care. Patients should be educated about the possible adverse effects of future cocaine use. Extensive skin involvement and necrosis may need surgical debridement and skin grafting. As happened in our patient, in extreme cases that involve extensive necrosis, amputation may be required to contain necrosis and infection.

## Conclusions

Adulteration of cocaine with levamisole is widely prevalent in United States. Levamisole may cause cutaneous vasculitis and neutropenia in people with cocaine use. History of cocaine use should be explored in patients presenting with a rash and neutropenia, and testing for levamisole exposure should be performed in selected patients. In cases with extensive involvement, this condition may lead to disfigurement by causing necrosis of skin, soft tissue, and cartilage.

### Informed consent

Written informed consent was obtained from the patient for publication of this case report and all accompanying images. A copy of the written consent is available for review by the Editor-in-Chief of this journal.

## Competing interests

None of the authors has any financial conflicts or competing interests to disclose.

## Authors’ contributions

NA conceived of the case report, performed the literature search, and drafted and revised the manuscript. TJ and RB acquired, analyzed, and interpreted the data. SN revised the manuscript. All authors read and approved the final draft.
